# Experience with non-vitamin K antagonists in the Netherlands: stories from both sides of the desk

**DOI:** 10.1007/s12471-019-01346-4

**Published:** 2019-11-19

**Authors:** T. A. C. de Vries, J. R. de Groot

**Affiliations:** 1 Heart Center, Department of Cardiology, Amsterdam University Medical Centres/University of Amsterdam, Amsterdam, The Netherlands; 2 Department of Cardiology, Rijnstate Hospital, Arnhem, The Netherlands

In the prevention of thromboembolic complications in patients with atrial fibrillation non-vitamin K antagonist oral anticoagulants (NOACs) are not only as effective as vitamin K antagonists (VKAs), but also have the advantage that they less frequently cause major bleeding events, in particular fewer intracranial haemorrhages [1–4]. Another important advantage of NOACs over VKAs: less variability in anticoagulant response, mainly caused by fewer food-drug and drug-drug interactions [5]. Due to this lower variability, a fixed dosing regimen applies to NOACs, obviating the need of routine monitoring of anticoagulant activity. This in contrast to treatment with VKAs [5]. However, this lack of monitoring may come with new challenges and downsides, namely more inappropriate dosing and non-adherence [6].

Thus, non-adherence to anticoagulants is likely to result in inappropriately low drug levels, and consequently, the cessation of the effect that they are intended for in the first place: to prevent thromboembolic complications [5, 6]. It is, therefore, relevant that we gain insight into the rate of thromboembolic and bleeding complications of NOACs in Dutch clinical practice, and the reasons why patients are non-adherent to their anticoagulant treatment. This issue of the *Netherlands Heart Journal* features two papers that address these topical subjects [7, 8].

Bennaghmouch et al. report on a prospective multi-centre survey study on self-reported adherence of 765 patients with atrial fibrillation (31.9% of all screened patients) to treatment of either NOACs (*n* = 376) or VKAs (*n* = 389). Unexpectedly, this study showed that self-reported non-adherence was more prevalent in VKA-treated patients than in those on NOACs (*n* = 95, 24.4% vs. *n* = 68, 18.1%; *p*-value 0.03) [7].

This observation might be related to practical issues intertwined with either type of anticoagulant, such as tablet shape/size or difficult dosing regimens. Thus, VKA-treated patients more frequently experienced such practical issues (*p* < 0.001): 63 (16.2%) of all VKA users commonly experienced practical issues, compared with 14 (3.7%) of those on NOACs [7].

Yet it is important to note that, in addition to those linked with surveys, two issues need to be considered. Firstly, the NOAC and VKA groups were not derived from the same population: users of VKAs were solely from a centre focussing on diagnostics located in Rotterdam, whereas the NOAC group was derived from a secondary/tertiary care hospital located in Nieuwegein [7].

Secondly, due to the differences in their offset of action and mechanism of producing an anticoagulant effect it’s unlikely that the reported non-adherence to both types of anticoagulants directly translates into a similarly reduced efficacy. All NOACs have a half-life between 8 and 15 hours and are direct inhibitors of either thrombin or factor Xa, whereas VKAs have a half-life between 11 hours (acenocoumarol) and 140 hours (phenprocoumon) and inhibit the hepatic synthesis of several clotting factors [5, 9]. Restoration of haemostasis in NOAC-anticoagulated patients, therefore, takes about 2 days (5 times the half-life), while for users of VKAs complete cessation of treatment is needed for at least 5 days or longer [5, 10]. Therefore, it may be more harmful to miss one dose of NOACs than of VKAs. Despite these considerations, no difference was observed in incidence of self-reported and verified adverse events, such as haematomas and bleeding events [7].

The second paper by De Veer et al. describes a prospective observational single-centre study, in which 799 patients were referred to a dedicated atrial fibrillation clinic and initiated on NOAC therapy, and provides us with a first report on the safety and efficacy of all 4 NOACs in Dutch clinical practice [8]. The distribution of the 4 NOACs was not even, but resembles the practice in the Netherlands: of the 799 patients, 507 (63.4%) were treated with rivaroxaban, and 182 (22.8%), 58 (7.3%), and 52 (6.6%) with apixaban, dabigatran, and edoxaban respectively [8].

Overall, these patients experienced 1.2 strokes and 1.8 major bleeding events per 100 patient-years during 1.7 years of follow-up, which is similar to the rate observed in the phase III randomised controlled trials [1–4], and other real-world studies [11–13]. The study underlines that use of NOACs in unselected Dutch patients with atrial fibrillation is safe and efficacious, although due to the lack of a control arm on VKA the differential effect of NOACs compared with VKA cannot be established. Of note, as no standardised follow-up was implemented, it cannot be excluded that safety or efficacy endpoints, in particular non-major event, may have been missed [8].

Aside from that, the authors also focus on discontinuation rates. In total 132 of 799 patients (16.5%) permanently discontinued their NOAC therapy, whereas in the phase III randomised controlled trials, such rates ranged between 21% and 25% during approximately 1.9 years of follow-up [8].

Importantly, most patients (*n* = 61, 46.2%) discontinued NOAC use because of end of treatment. Examples include those patients with a CHA_2_DS_2_-VASc score of 0 who underwent successful elective cardioversion [8]. As such patients on temporary NOAC therapy were not included in the phase III trials [1–4], it is unlikely that the rates of permanent discontinuation in Dutch clinical practice will have a clinically relevant impact on the overall relative treatment effect of NOACs compared with VKAs. Again, the proportion of patients who discontinued treatment may have been underestimated due to the non-standardised follow-up [8].

With the NOACs emerging as the preferred stroke prevention in patients with atrial fibrillation, we now have drugs available that overcome several shortcomings of VKAs. However, they may come with novel issues such as underdosing or non-adherence, which may undermine the purpose of the therapy: reducing the risk of ischaemic strokes. The contributions on this topic in the current issue of the *Netherlands Heart Journal *give relevant insights into the use of all 4 NOACs in Dutch clinical practice from both sides of the desk. These papers indicate that safety and efficacy of NOACs, as well as self-reported adherence, seem to fall well within the range of expectation, and may even be better than for VKAs [7, 8].
